# Exercise in Heart Disease

**Published:** 1902-07-05

**Authors:** 


### Exercise in Heart Disease.

Among the many changes which have taken place during the past twenty years in the methods adopted in the treatment of heart disease, few have been more marked than those which depend on the recognition of the fact that better results are to be obtained by exercise than by rest. The facts having been ascertained that the maintenance of health, and indeed in many cases of life, in the presence of disease of the heart depends on the development of compensatory cardiac hypertrophy, and that such hypertrophy tends to break down when exposed to strain, men were led in times gone by to the apparently logical conclusion that the heart ought at all hazards to be saved from effort lest its compensatory hypertrophy should be endangered. Hence for many years it was the custom to treat cardiac cases on such a system of coddling and avoidance of exertion as to leave the heart at the mercy of any sudden call for increased effort which might be made upon it by the various vicissitudes or accidents of life.

During recent years, however, a great change has come over the view taken of cardiac therapeutics, and especially of the question of exercise in heart cases. It has become increasing obvious that the power of the cardiac, as indeed of any other muscle, has a certain relation to the range within which it is accustomed to be exerted, and that in most cases (putting on one side cases of contracted kidney) when failure of compensation does ultimately occur it arises not from the heart being unable to meet the demands made upon it by the ordinary daily routine, but from its being exposed to some extra strain, arising from some intercurrent illness or unavoidable exertion. Thus it became clear that if cardiac patients were to be at all safe their hearts must be trained and accustomed to do a good deal more than meet the calls made upon them by the strictly ordered and carefully regulated mode of life which used to be considered the only state of existence proper to such cases.

Oertel, with his *terrain cure*, was in its early days the great spokesman of the new method, and since then exercises of many kinds have attained a great popularity in the treatment of heart disease. It is to be feared, however, that even now it is not as well recognised as it should be that the real and the great utility of exercise lies in what one may call the management of heart cases rather than in the treatment of heart disease. Useful as are the special exercises which form part of the Nauheim treatment, that is only the beginning of the utility of "exercise" taken in its wider meaning, for it is when the patient considers himself cured, when in other words the cardiac compensation has become perfect for its ordinary needs, that the great necessity arises for entering upon a prolonged régime, one of the main factors of which is the taking of proper exercise, with the object of maintaining such compensation as exists and developing it in such degree as to meet the unavoidable emergencies of life.

In arranging such a course of exercise one has to seek some form of effort which can be graduated to a nicety, which shall make a proper call upon the respiratory muscles, and in which there shall be no temptation to excess, and there is probably no form so useful and convenient as gentle walking up hill. It is taken in the open air, it can be obtained at any time which is convenient, without the aid of an assistant, and it is capable of being regulated with the utmost nicety. It may appear at first sight that some form of exercise in which the effort should be less exclusively confined to the lower extremities might be more advantageous, but as a fact one of the great advantages of Oertel's *terrain cure*, lies in the fact that by it the circulation is specially assisted (as the lymph and venous circulation always is assisted by muscular contractions) just in that part where it more particularly tends to fail. Many other forms of exercise are at our disposition, but most of them have drawbacks. Rowing has been highly spoken of by some, but mere paddling falls too exclusively upon the muscles springing from the chest, while proper rowing is rather beyond what one can safely endure in most cardiac cases. For home use on wet days, however, some form of portable rowing apparatus may be found very advantageous. If one could be quite sure of never getting into an awkward corner in which both nerve and muscle may be momentarily strained, cycling would be very good. The cyclist, however, is always at the mercy of the unexpected, and, if for this reason alone, bicycling is barred to many. As for golf, tennis, and such like games, involving sudden spurts of effort, they also are better left alone, as are all games into which the element of competition largely enters. But, whatever the form of exercise, the patient should be kept under pretty constant observation. The great danger is lest he should forget that he has a heart, and should develop a fondness for the exercise for its own sake. A cardiac patient must never try to become an athlete. The object of all his exercise is to keep the power of his heart a little above the daily demands upon it, and thus to insure the possession of a certain amount of reserve. Unfortunately few patients are content with this.

